# Mitotic cell death caused by follistatin-like 1 inhibition is associated with up-regulated Bim by inactivated Erk1/2 in human lung cancer cells

**DOI:** 10.18632/oncotarget.6729

**Published:** 2015-12-22

**Authors:** Kieun Bae, Kyoung Eun Park, Jihye Han, Jongkwang Kim, Kyungtae Kim, Kyong-Ah Yoon

**Affiliations:** ^1^ Research Institute and Hospital, National Cancer Center, Goyang, Gyeonggi, Korea; ^2^ College of Veterinary Medicine, Konkuk University, Gwangjin-gu, Seoul, Korea

**Keywords:** FSTL1, lung cancer, apoptosis, Bim, Erk1/2

## Abstract

Follistatin-like 1 (FSTL1) was identified as a novel pro-inflammatory protein showing high-level expression in rheumatoid arthritis. The protective effect of FSTL1 via the inhibition of apoptosis was reported in myocardial injury. However, the functional mechanism of FSTL1 in cancer is poorly characterized, and its proliferative effects are ambiguous. Here, we examined the effects of FSTL1 on cellular proliferation and cell cycle checkpoints in lung cancer cells. FSTL1 inhibition induced the cellular portion of G2/M phase in human lung cancer cells via the accumulation of regulators of the transition through the G2/M phase, including the cyclin-dependent kinase 1 (Cdk1)-cyclin B1 complex. An increase in histone H3 phosphorylation (at Ser10), another hallmark of mitosis, indicated that the knockdown of FSTL1 in lung cancer cells stimulated a mitotic arrest. After that, apoptosis was promoted by the activation of caspase-3 and -9. Protein level of Bim, a BH3 domain-only, pro-apoptotic member and its isoforms, BimL, BimS, and BimEL were up-regulated by FSTL1 inhibition. Degradation of Bim was blocked in FSTL1-knockdown cells by decreased phosphorylation of Bim. Increased BimEL as well as decreased phosphorylated Erk1/2 is essential for cell death by FSTL1 inhibition in NCI-H460 cells. Taken together, our results suggest that the knockdown of FSTL1 induces apoptosis through a mitotic arrest and caspase-dependent cell death. FSTL1 plays the important roles in cellular proliferation and apoptosis in lung cancer cells, and thus can be a new target for lung cancer treatment.

## INTRODUCTION

Follistatin-like 1 (FSTL1) was originally isolated as a TGF-β stimulating clone (TSC-36) and was found to inhibit TGF-β superfamily proteins [1]. FSTL1 is a pro-inflammatory protein secreted under inflammatory conditions (e.g., arthritis) that induces the up-regulation of inflammatory cytokines [2, 3]. Furthermore, FSTL1 was also known as a useful biomarker of cardiovascular disease including myocardial infarction and acute coronary syndrome [4, 5]. In the context of these diseases, FSTL1 is a cardiac-secreted factor with protective effects against cellular damage. The anti-apoptotic and -inflammatory effects of FSTL1 in the heart are mediated by the modulation of AMP-dependent protein kinase and bone morphogenic protein-4. Furthermore, FSTL1-deficient mice show impaired tracheal formation and alveolar epithelial cell differentiation, leading to postnatal lethality due to respiratory failure [6]. It has also been shown that FSTL1 functions as a mesenchymal factor in the determination of epithelial cell fate in the oviduct [7].

The various functions of FSTL1 suggest that it plays important roles in epithelial carcinoma. Recently, FSTL1 was suggested as a triggering molecule for bone metastasis with the inhibited metastatic efficacy by FSTL1 blocking [8]. However, the role of FSTL1 in cancer remains controversial. FSTL1 overexpression has been reported in glioblastoma and is a hallmark of a poor prognosis [9]. In contrast, FSTL1 exerts tumor-suppressive actions in ovarian and endometrial carcinogenesis, and it is down-regulated in metastatic clear-cell renal-cell carcinoma [10, 11]. The expression pattern of FSTL1 is not consistent in lung cancer when we compared several data sets from the Oncomine database (Supplementary Figure S1). However, the essential role of FSTL1 in mouse lung development as shown by FSTL1-deficient mice makes it plausible to study the role of FSTL1 in lung cancer cells.

In this study, we investigated the effects of FSTL1 on cell cycle and apoptosis in lung cancer cells. A cell cycle arrest and the induction of apoptosis through Bim accumulation were observed in FSTL1-knockdown cells. This is the first report to suggest the potential therapeutic effect of FSTL1 blockade in lung cancer.

## RESULTS

### FSTL1 blocking induced cell death in non-small cell lung carcinoma (NSCLC) cells

**Figure 1:**
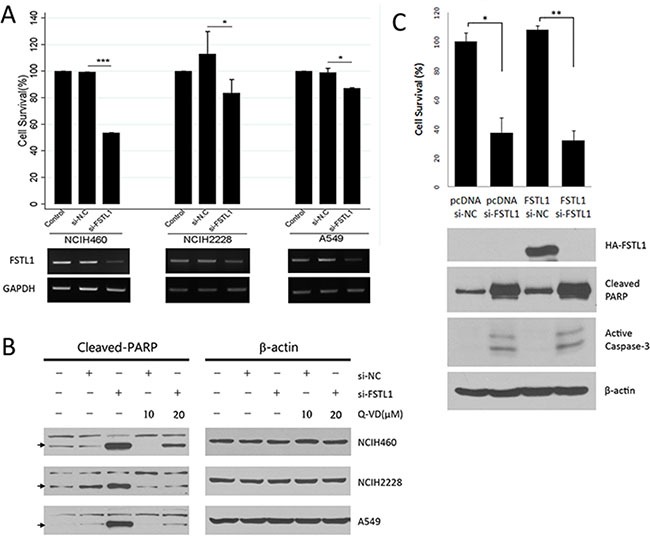
Cell death was induced in FSTL1-knockdown NSCLC cells (**A**) A total cell number was analyzed after FSTL1 inhibition in NCI-H460 (large cell carcinoma), H2228 (lung adenocarcinoma), and A549 (lung carcinoma) cells. Data are expressed as means ± SD from three repeated experiments and statistical significant differences are marked with *(*p* < 0.05), and ***(*p* < 0.0005), respectively. Inhibition of FSTL1 expression by siRNA was confirmed by RT-PCR. Glyceraldehyde-3-phosphate dehydrogenase (GAPDH) was used as control. (**B**) Protein expression of cleaved-PARP was compared by western blotting at 48 hours after transfection of siRNA. Transfected cells with siRNAs were treated with a pan-caspase inhibitor, quinolyl-valyl-aspartate-OPh (Q-VD-Oph) to examine the effects of caspase inhibition. β-actin served as a loading control. (**C**) Cell number and apoptotic proteins were analyzed in transfected cells with FSTL1 expression vector (FSTL1) or empty vector (pcDNA), followed by siRNA treatment. Statistical differences are marked with *(*p* < 0.05), and **(*p* < 0.005), respectively.

FSTL1 was inhibited in NSCLC cells (NCI-H460, NCI-H2228, and A549) by the transfection of a siRNA specific for FSTL1. There was a significant reduction in the number of cells transfected with siRNA for FSTL1 (si-FSTL1) compared with negative control siRNA (si-NC) transfected cells. Among the cell lines, NCI-H460 showed the highest rate of cell death (≥ 46.4%), whereas cell lines A549 and NCI-H2228 showed a moderate degree of cell death (≥ 12.9 and 18.4%, respectively) (Figure 1A). In addition, FSTL1 knockdown resulted in a significant increase in cell death in lung cancer cells as indicated by PARP cleavage determined by western blotting (Figure 1B). We subsequently used NCI-H460 cells, which showed the highest rate of cell death, to evaluate FSTL1 knockdown-induced cell death. FSTL1 blocking induced cell death and activated caspase-3 were also confirmed in NCI-H460 cells that were transfected with FSTL1 expression vector before knockdown (Figure 1C). To exclude the off-target effect of single siRNA, we tested three different siRNAs for FSTL1. The effect on cell death shown by PARP cleavage was confirmed in all kinds of siRNAs for FSTL1 (Supplementary Figure S2).

### FSTL1 knockdown induced a G2/M arrest and cyclin-Cdk up-regulation

**Figure 2:**
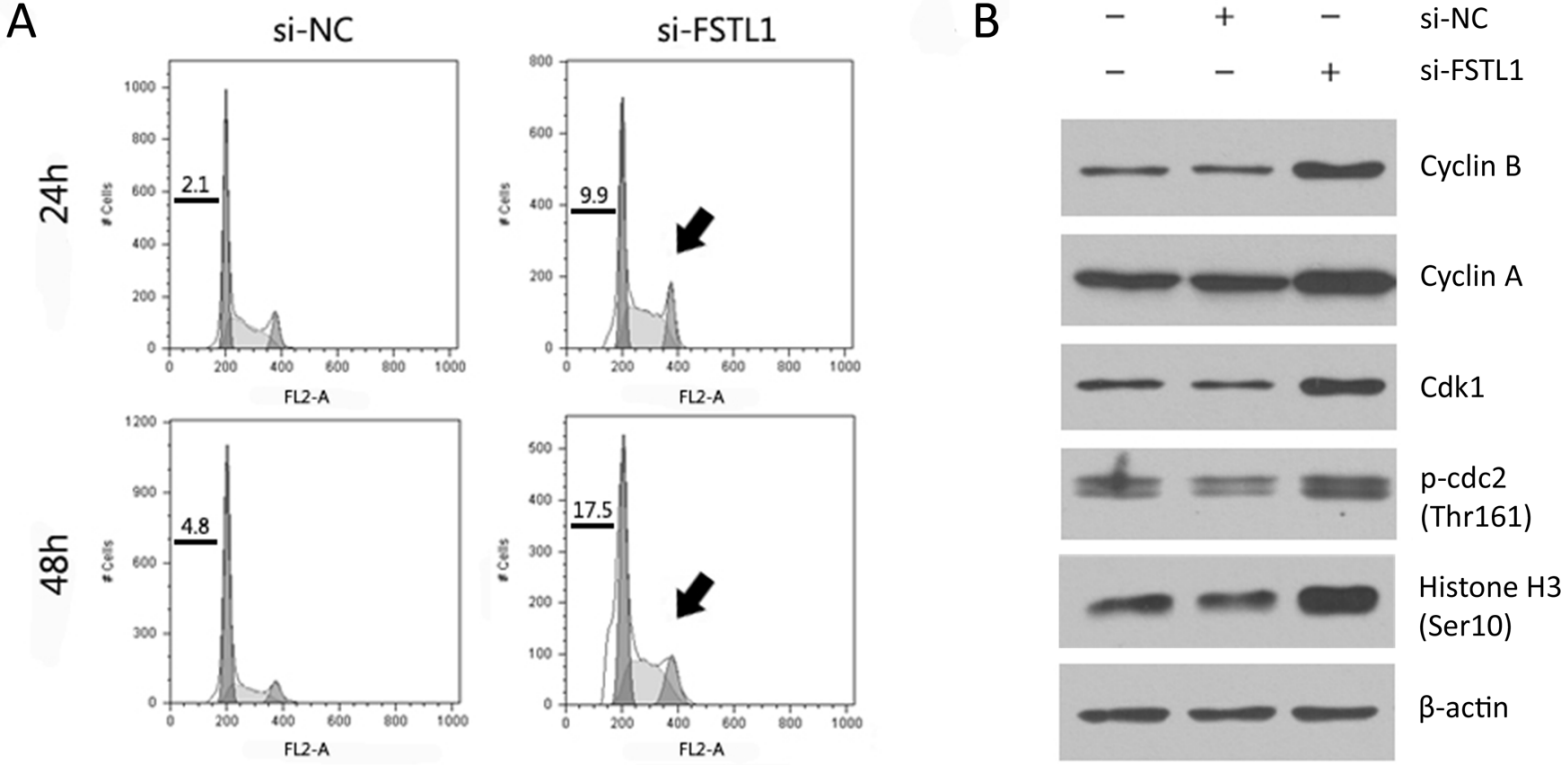
FSTL1-knockdown induced G2/M arrest and cyclin B1-Cdk1 up-regulation (**A**) Cell cycle was analyzed in NCI-H460 cells transfected with siRNA-FSTL1 at the indicated time points. Sub-G1 fraction was shown as the number (%) and the increased G2/M phase was indicated by an arrow. (**B**) Western blotting analysis displayed the protein level of cyclin B1, cyclin A, Cdk1, and phohsphorylated-Cdc2 (Thr161) in NCI-H460 cells.

We next examined the effect of FSTL1 knockdown on cell cycle progression. After transfection of siRNA-FSTL1, the proportion of G2/M phase cells was increased in NCI-H460 cells (Figure 2A). To understand the mechanism by which the knockdown of FSTL1 induces G2/M arrest, we measured the levels of several key proteins that regulate the cell cycle, including cyclin B1, cyclin A, Cdk1, and phohsphorylated-Cdc2 (Thr161). As shown in Figure 2B, the protein levels were markedly increased in FSTL1-knockdown cells. The regulators of the transition through the G2 phase to mitosis, including the Cdk1, cyclin B1 were dysregulated after the knockdown of FSTL1 in NCI-H460 cells. Increased phosphorylated histone H3 protein level indicated that the knockdown of FSTL1 in NCI-H460 cells stimulated an arrest in mitosis, but not in G2.

**Figure 3:**
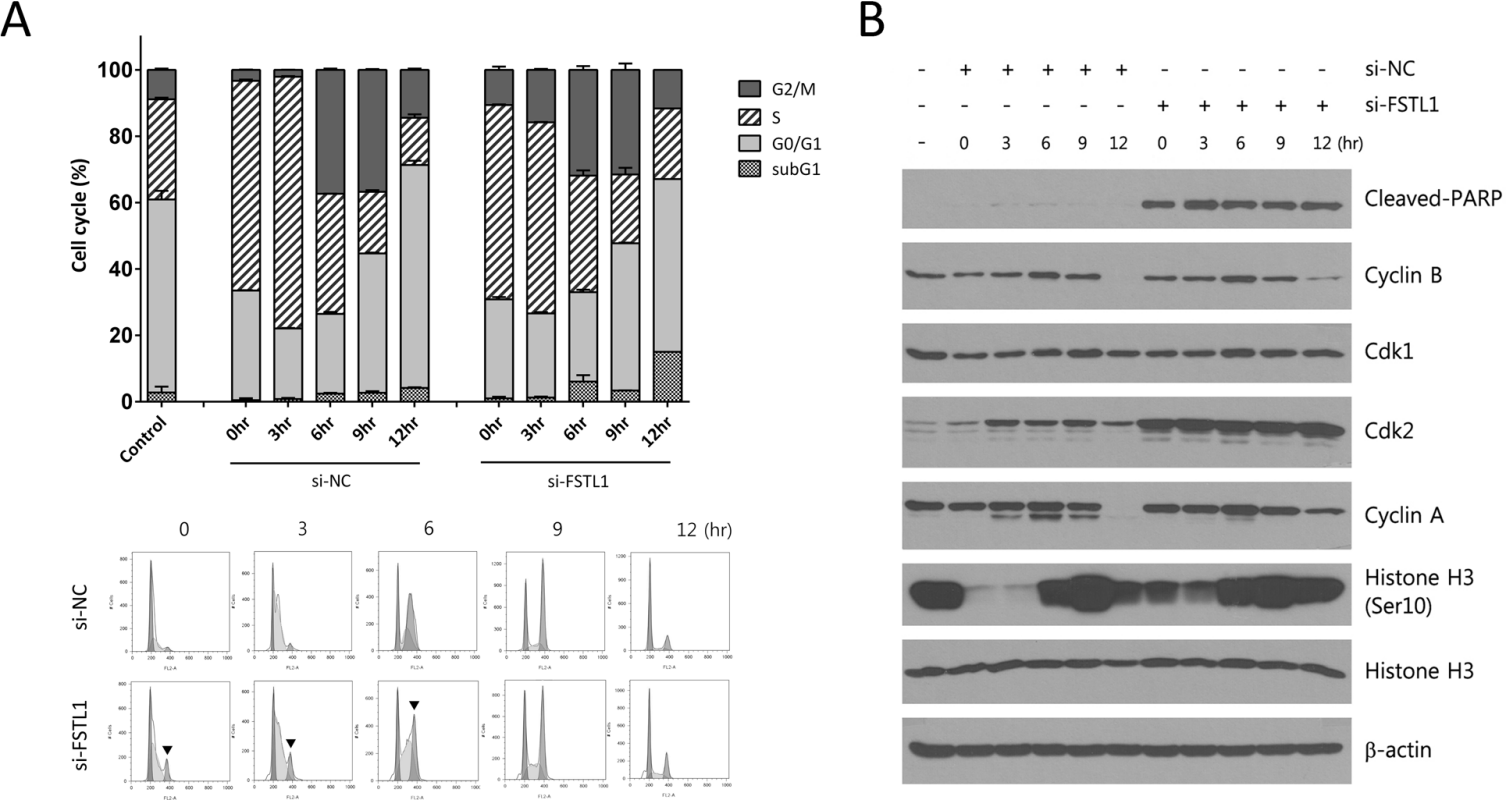
FSTL1 inhibition by siRNA induced mitotic arrest NCI-H460 cells transfected with siRNA for *FSTL1* or negative control were synchronized in G1 by a double thymidine block, and then released into the cell cycle for the times indicated. (**A**) Cell cycle was analyzed at the indicated time points after a double thymidine block. Each bar in the graph indicated average cell cycle distribution from triplicated experiments. Representative DNA histograms were demonstrated and the retained cells in G2/M phase were indicated by arrowheads. (**B**) Cell cycle-related proteins, cyclin B1, cyclin A, Cdk1, Cdk2 and histone H3 phosphorylated at Ser10, were evaluated by western blotting. Cleaved PARP was examined as cell-death indicator. β-actin was used for loading control.

To validate these observations, cells were synchronized at G1/S phase using a double thymidine block, then released into the cell cycle as determined by flow cytometric analysis. FSTL1 blocking attenuated synchronization at G1/S phase and preserved G2/M peak after cells were released from thymidine block (Figure 3A). The cells transfected with a negative control siRNA entered mitosis at around 9 hours after the thymidine block, as determined by the maximum level of mitotic phosphorylation of histone H3 at Ser10. However, the FSTL1-knockdown cells showed retained upregulation of Cdk2, phosphorylated Histone H3, and cleaved-PARP (Figure 3B). The results suggested FSTL1 blocking induced dysregulation of cell cycle.

### FSTL1 inhibition triggers apoptosis by caspase activation

**Figure 4:**
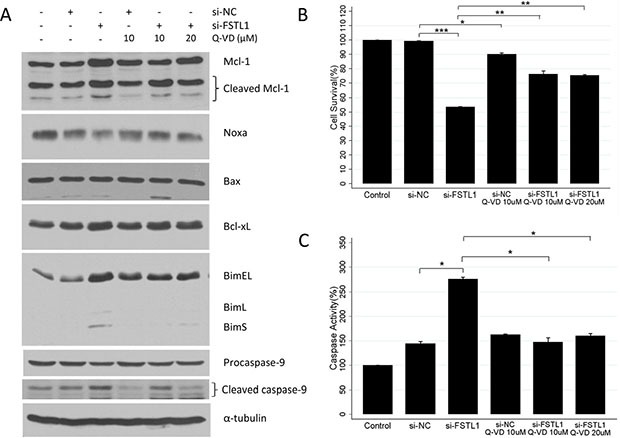
FSTL1 inhibition induced caspase-dependent apoptosis NCI-H460 cells were treated with the pan-caspase inhibitor Q-VD-Oph (10, 20 μM) or dimethyl-sulfoxide (DMSO) after transfection of siRNAs. (**A**) Protein expression was examined by western blotting for apoptosis associated proteins including Mcl-1, Noxa, Bax, Caspase-9 and Bim. (**B**) Number of viable cells was determined after treatment. Statistical significant differences are marked with *(*p* < 0.05), **(*p* < 0.005) and ***(*p* < 0.0005), respectively. (**C**) Apoptosis was quantified by caspase-3 activity in cytosolic extracts. Caspase-3 activity was measured on cytosol lysates using the level of chromophore p-nitroaniline (pNA) after cleavage from the labeled 200 μM DEVD-pNA substrate. Statistical significant differences are marked with *(*p* < 0.05).

Transfection of siRNA for FSTL1 significantly induced cell death that could be rescued by treatment with 10 or 20 μM of a pan-caspase inhibitor, quinolyl-valyl-aspartate-OPh (Q-VD-Oph). To examine the mechanism of cell death in mitosis caused by the knockdown of FSTL1, we assessed the levels of apoptotic proteins. As shown in Figure 4A, FSTL1 blocking had no effect on Bax, Bcl-xL, and Noxa level. However, FSTL1 inhibition in NCI-H460 cells significantly increased the levels of BimEL, as well as the BimL and BimS isoforms, and the cleaved forms of Mcl-1. Increase of BimEL level, and Mcl-1 cleavage were determined to be in caspase-dependent manner. Caspase-9 was also activated in FSTL1-knockdown cells. Inhibition of caspases with Q-VD-Oph avoided FSTL1-knockdown induced cell death as demonstrated in Figure 4B. The activity of caspase-3 in the FSTL1-knockdown cells was greatly increased compared to that in the controls, and this activation was inhibited by treatment with a pan-caspase inhibitor, Q-VD-Oph (Figure 4C). These results indicated apoptosis in FSTL1-knockdown cells required the activation of caspase. In addition, they suggest a specific function for the accumulated Bim isoforms in the regulation of apoptosis.

### Apoptosis induced by FSTL1 blocking is associated with inhibited phosphorylation of Bim

**Figure 5:**
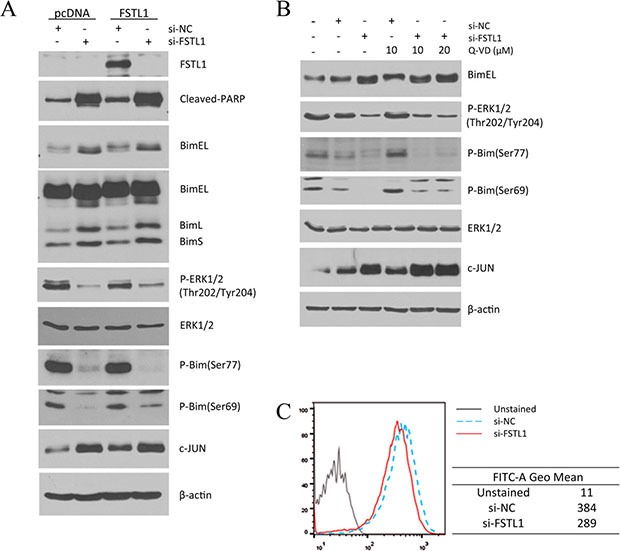
FSTL1-knockdown induced apoptosis was associated with increased Bim by decreased phosphorylation The effect of FSTL1 inhibition enhanced the levels of three isoforms of BH3-only Bim protein, including BimEL, BimL, and BimS. (**A**) Protein expression level was analyzed in transfected cells with FSTL1 expression vector (FSTL1) or empty vector (pcDNA), followed by siRNA treatment. (**B**) The effects of caspase inhibition was tested by treatment of a pan-caspase inhibitor, Q-VD-Oph in siRNA transfected cells. Bim isoforms, phosphorylated Bim, and Erk1/2 level were examined in FSTL1-knockdown cells by western blot analysis. β-actin was used for loading control. (**C**) Phosphorylated-Erk1/2 activation was assessed by flow cytometric analysis. Histogram shows down-regulation of phosphorylated Erk1/2 (Thr202/Tyr204) in FSTL1-knockdown cells relative to negative control (si-NC transfected cells).

Bim is a BH3 domain-only, pro-apoptotic, Bcl-2 family member that is known to potently bind to and inhibit anti-apoptotic proteins like Mcl-1. Among three Bim isoforms, BimL and BimS are reported as the stronger apoptotic proteins compared to BimEL. We found that three isoforms of Bim were upregulated by FSTL1 knockdown as shown in Figure 5A. We detected the increased and shifted band of BimEL protein in FSTL1-knockdown cells using western blotting. Interestingly, phosphorylation of Bim at Ser69 and Ser77 were decreased by FSTL1 blocking (Figure 5A and 5B). To examine the activation of Erk1/2 that regulates Bim by phosphorylation, phosphorylation of Erk1/2 at Thr202/Tyr204 was measured by western blot and phosphor-flow assay. Decreased phosphorylated Erk1/2 protein was detected in FSTL1-knockdown cells as shown in Figure 5B and 5C. However, FSTL1 blocking did not alter the protein level of Erk1/2. FSTL1 blocking inhibited Erk1/2 activation that resulted in the accumulated Bim isoforms due to suppressed Bim phosphorylation.

## DISCUSSION

In this study, we found that apoptosis induced by FSTL1 inhibition was associated with up-regulated BimEL by decreased phosphorylation through Erk1/2 inactivation. FSTL1, also known as TSC-36, is a well-known pro-inflammatory molecule. Due to its multiple functions, defining the role of FSTL1 in cancer has been a challenge. Recent results from Fstl1-deficient mice, which displayed impaired lung development and alveolar maturation, motivated us to study the relationship between FSTL1 and lung cancer.

Our data suggest interplay between the mitotic arrest and apoptosis through FSTL1. FSTL1 inhibition induced a mitotic arrest via the accumulation of cyclin B1-Cdk1 complexes. It is unclear whether FSTL1 itself can regulate cell cycle progression; however, FSTL1 inhibition could cause a cell cycle arrest by generating an imbalance between cell cycle regulatory proteins. The accumulation of cyclin B1-Cdk1 complexes is thought to be the major cause of cell cycle damage. Constant cyclin B1-Cdk1 expression driving both a mitotic arrest and mitotic cell death has been reported in several studies. [12]. Cdk1/cyclin B has been shown to phosphorylate and inactivate anti-apoptotic members of the Bcl-2 protein family, including Bcl-xL, Bcl-2 and Mcl-1, which oppose apoptosis by binding to the pro-apoptotic members and neutralizing their activity [13–15].

Apoptosis was followed by Caspase-3 and -9 activation in FSTL1-knockdown cells. Caspase-dependent Mcl-1 cleavage after FSTL1 inhibition was considered as pro-apoptotic event with Mcl-1 upregulation. Mcl-1 has a critical function in the control of apoptosis as anti-apoptotic protein. However, it was recently shown that Mcl-1 cleavage products have a pro-apoptotic function via a feed-forward amplification loop [16, 17]. Therefore, the accumulated Mcl-1 cleavage products detected after FSTL1 inhibition could amplify the apoptotic cascade. We examined the protein level of Noxa, a well-known pro-apoptotic molecule. Protein level of Noxa was not changed in apoptotic cells. The interaction among Noxa, Mcl-1, and Bim was reported to be important to determine cell death [18–20]. However, we could not detect the changed level of Noxa by FSTL1-knockdown. Instead of Noxa, upregulation of c-Jun was resulted in siFSTL1 induced apoptotic cells. Recently, Bortezomib induced Mcl-1 cleavage that induced apoptosis via c-Jun upregulation [21]. Although recent studies reported c-Jun mediated cell death, the interaction of c-Jun, Mcl-1, and FSTL1 should be investigate in further study [22–24].

In our study, FSTL1 blocking inhibited the phosphorylation of Bim protein. Bim (Bcl-2 interacting mediator of cell death) is one of pro-apoptotic ‘Bcl-2 homology 3-only’ proteins that promote cell death. Bim shows three major isoforms including Bim short (BimS), Bim long (BimL), and Bim extra-long (BimEL) by alternative splicing [25]. Bim protein is always overexpressed in epithelial cancers and each isoforms showed different apoptotic potencies [26]. Regulation of Bim expression by Erk signaling pathway is a critical step in the induction of apoptosis. Phosphorylation of Bim is an important mechanism to regulate its activity and function. Phosphorylated BimEL by Erk1/2 promoted its proteasomal degradation and inhibit apoptotic cell death [27]. Our data showed FSTL1 blocking decreased phosphorylated BimEL on serine 69 and 77 as well as phosphorylation of Erk1/2. Increased protein level of Bim promoted caspase-dependent apoptosis in FSTL1 knockdown cells. Oshima *et al.* demonstrated that increased Erk and Akt phosphorylation in FSTL1 overexpressed cardiac myocytes [5]. The authors reported the protective action of FSTL1 from hypoxia-induced apoptosis through the activated Akt and Erk signaling systems. In our study, we demonstrated inactivated Erk1/2 by FSTL1 knockdown that resulted in downregulated phosphorylation of Bim. To our knowledge, this is the first report on the association of FSTL1 with pro-apoptotic protein Bim. Taken together, our results suggest that FSTL1 plays an important role in maintaining cell cycle transitions and its inhibition promotes apoptotic event in lung cancer. Thus, FSTL1 could be a potential candidate therapeutic target for lung cancer.

## MATERIALS AND METHODS

### Cell culture and cell cycle synchronization

A549, NCI-H460, and NCIH2228 lung cancer cells were obtained from the ATCC (Manassas, VA, USA) and maintained in RPMI1640 containing antibiotics and 10% fetal bovine serum at 37°C with 5% CO_2_ in a humidified atmosphere. NCI-H460 cells were synchronized by the double thymidine block method. Briefly, cells (1.5 × 10^5^) in a 6-well plate were incubated in medium containing 2 mM thymidine (Sigma-Aldrich, St. Louis, MO, USA) for 16 hours, released into normal medium for 9 hours, and then incubated for 16 hours in medium containing 2 mM thymidine.

### Transfection of siRNAs or plasmids

Specific siRNAs for silencing FSTL1, and a negative control siRNA (si-NC) that showed no significant sequence similarity to human gene sequences, and lipofectamine 2000 reagent were purchased from Ambion^®^ Life Technologies (Carlsbad, CA, USA). Expression vector of FSTL1 was constructed by subcloning of full sequence of FSTL1 open reading frame tagged with hemagglutinin (HA) into pcDNA3.1 (+) vector (Invitrogen, Carlsbad, CA, USA). For transient expression of FSTL1, pcDNA3.1-FSTL1 construct (HA-FSTL1) and empty vector (pcDNA) were transfected into cells with lipofectamine 2000 reagent followed by the further transfection with siRNAs.

### Cell counting and cell cycle analysis

The total cell number and cell viability were assessed using a Scepter handheld automated cell counter (Millipore, Billerica, MA, USA) according to the manufacturer's recommendations. To determine the number of viable cells, dye exclusion test was used by applying 0.4% trypan blue. The cell cycle was analyzed by flow cytometry. Cells were harvested, washed in PBS, and resuspended in 70% ethanol. After incubation at 4°C for 60 min, the cells were resuspended in propidium iodide (50 μg/ml) and RNase A (10 μg/ml) and incubated for 30 min at 37°C. Propidium iodide, trypan blue, and RNase A were purchased from Sigma Chemical (St. Louis, MO, USA). The cell cycle was assessed by flow cytometry (FACSCalibur II; BD Diagnostics, Sparks, MD, USA) and analyzed using the FlowJo software, version 5.7.2 (Tree Star Inc., Ashland, OR, USA). All experiments were performed in triplicate and representative data were shown in Figures.

### Western blot analysis

Cells were harvested, washed with PBS, and a lysate was prepared in sample buffer (125 mM Tris-HCl, pH 6.8, 20% glycerol, 4% sodium dodecyl sulfate [SDS], and 10% 2-mercaptoethanol) on ice for 20 min. The solution was cleared by centrifugation at 10,000 rpm for 15 min at 4°C. The amount of protein was measured using a Qubit Protein Assay Kit (Invitrogen). Cytosolic fractions were prepared using Cytoplasmic extraction reagent (BioVision Inc., Milpitas, CA, USA). After incubation for 15 min on ice and centrifugation at 13,000 rpm for 1.5 min at 4°C, the cytosolic fraction was recovered. Cytosolic protein was quantitated using the Bradford method (Dc Protein Assay; Bio-Rad, Hercules, CA, USA). Equal protein concentrations were mixed with lysis buffer, separated by 10 or 12% SDS-polyacrylamide gel electrophoresis, and electro-transferred to Immobilon-P polyvinylidene difluoride or nitrocellulose membranes (Millipore). The membranes were blocked for 1 hour in 5% skim milk in Tris-buffered saline with Tween-20 (TBST; 10 mM Tris-HCl, pH 8.0, 150 mM NaCl, and 0.5% Tween-20). The membranes were incubated overnight at 4°C then washed with TBST, followed by a 2-h incubation with secondary antibodies. After a final wash with TBST, the membranes were developed by enhanced chemiluminescence according to the manufacturer's instructions and exposed to photographic film. Polyclonal antibodies against cleaved-PARP, HA, cyclin B1, cyclin A, Cdk1, Cdk2, phosphorylated-Cdc2 (Thr161), histone H3, caspase-9, Mcl-1, Bcl-xL, Bax, Bim, phosphorylated-Erk1/2 (Thr202/Tyr204) and horseradish peroxidase-linked secondary antibodies were obtained from Cell Signaling Technology (Danvers, MA, USA). Noxa and α-tubulin were purchased from Santa Cruz Biotechnology (Santa Cruz, CA, USA). Anti-β-actin and -histone H3 (phosphorylated-Ser10) were obtained from Abcam (Cambridge, MA, UK). Electrochemiluminescence (ECL) reagent was from Amersham Life Science (Buckinghamshire, UK). The results of western blotting analysis were demonstrated with representative examples from three independent experiments.

### RT-PCR and quantitative real-time PCR

RNA level of FSTL1 was examined by RT-PCR using specific primers for human FSTL1 (forward primer, 5′-CCTGTGTGTGGCAGTAATGG-3′ and reverse primer, 5′-TCAGGAGGGTTGAAAGATGG-3′). Total RNA was isolated with TRIzol reagent (Life technologies) according to manufacturer's guide. From total RNA, cDNA was synthesized with reverse transcriptase and random hexamer (Invitrogen). Real-time RT-PCR was performed with PowerUp Sybr green master mix (Applied biosystems) and specific primers (forward primer, 5′-CGATGGACACTGCAAAGAGA-3′ and reverse primer, 5′- CCAGCCATCTGGAATGATCT-3′) using Quantstudio7 Flex real-time PCR system (Thermo Fisher Scientific Inc., Waltham, MA, USA).

### Caspase-3 activity assay

Caspase-3 activity was determined by measuring the level of chromophore p-nitroaniline (pNA) after cleavage from the labeled substrate DEVD-pNA using commercially available kits according to the manufacturer's protocols. The Caspase-3/CPP32 Colorimetric Assay Kit was obtained from BioVision Inc. (Milpitas, CA, USA). Caspase-3 activity was determined by comparing the level of substrate cleavage in transfected and/or treated samples versus that in controls. The NCI-H460 cell line was treated with a pan-caspase inhibitor, quinolyl-valyl-aspartate-OPh (Q-VD-Oph) (10 or 20 μM, MP Biomedicals, Santa Ana, CA, USA), for 48 hours.

### Flow cytometric analysis of phosphorylated Erk1/2

Harvested cells were fixed in Phosflow™ Lyse/Fix buffer (BD Biosciences) for 10 min at 37°C and permeabilized with buffer containing 90% cold methanol. For determination of Erk1/2 phosphorylation, permeabilized cells were incubated with anti-phosphorylated-Erk1/2 (Thr202/Tyr204) antibody (Cell Signaling Technology, Inc.) for 1 hour at room temperature. After washed with Pharmingen™ Stain buffer, cells were stained with goat anti-rabbit antibody conjugated AlexaFluor 488 for 1 hour at room temperature. Fluorescence-labeled cells were measured by FACSVerse flow cytometer (BD Biosciences) and analyzed using FlowJo software.

### Statistical analysis

Statistical significance was tested by unpaired *t*-Test for group comparison using the R software system. Significant differences are marked with *(*p* < 0.05), **(*p* < 0.005), and ***(*p* < 0.0005) on figures, respectively.

## SUPPLEMENTARY MATERIALS FIGURES


