# Supplementary material for: Mitotic cell death caused by follistatin-like 1 inhibition is associated with up-regulated Bim by inactivated Erk1/2 in human lung cancer cells
Source: Oncotarget. 2015 Dec 22;7(14):18076–84. doi: 10.18632/oncotarget.6729 (PMC4951272; doi:10.18632/oncotarget.6729)
Supplement: Supplementary file 1 [file oncotarget-07-18076-s001.pdf]

Mitotic cell death caused by follistatin-like 1 inhibition is associated with up-regulated Bim by inactivated Erk1/2 in human lung cancer cells

Supplementary Materials

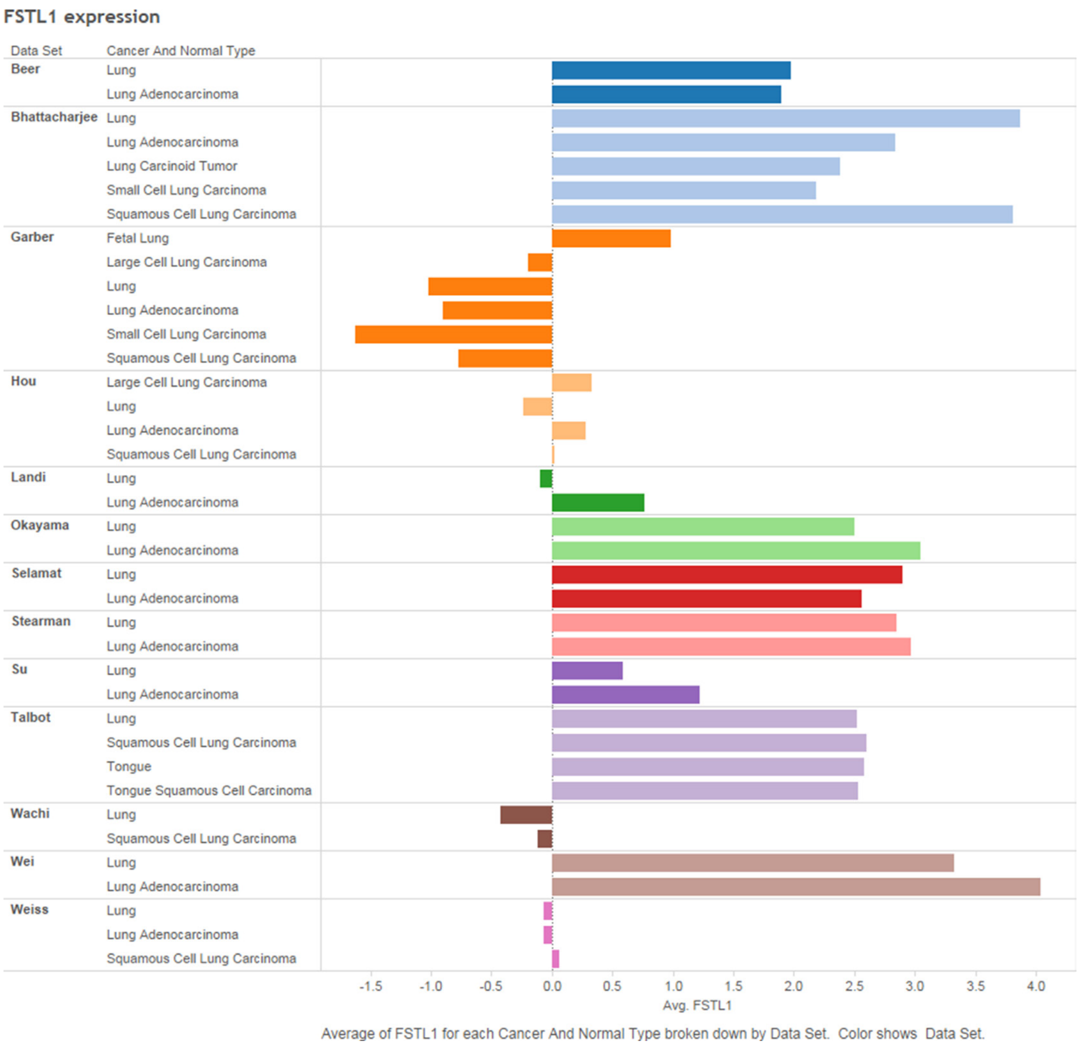

Supplementary Figure S1: FSTL1 expression in lung cancer was compared with data sets from Oncomine database. We compared FSTL1 gene expression in lung cancer using the Oncomine database with 13 data sets. Data sets on lung cancer were collected from the Oncomine database (http://www.oncomine.org). We compared FSTL1 expression in normal lung (lung) and different subtypes of lung cancer. Elevated FSTL1 expression in lung adenocarcinoma compared to normal lung tissue was demonstrated from six data sets. Publications for 13 studies that provided data were listed below [1–13].

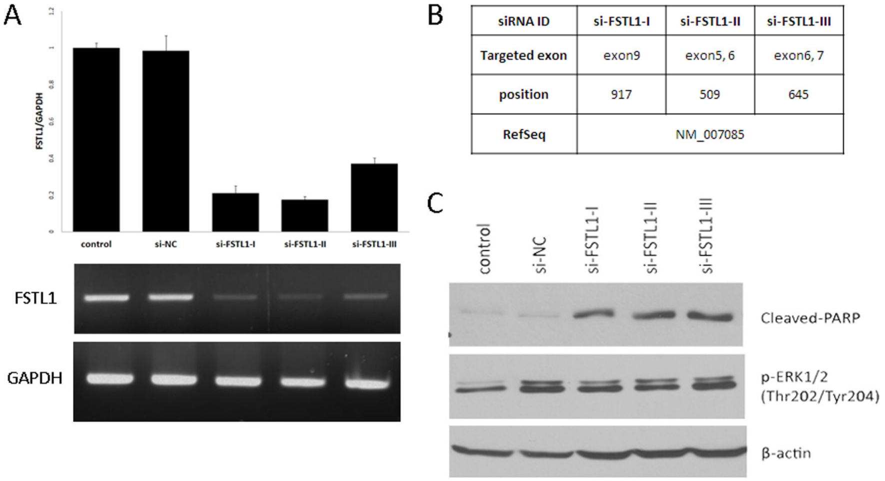

siRNA ID	si-FSTL1-I	si-FSTL1-II	si-FSTL1-III
Targeted exon	exon9	exon5, 6	exon6, 7
position	917	509	645
RefSeq	NM_007085		

Supplementary Figure S2: FSTL1 knockdown with siRNAs inhibited FSTL1 expression and induced cleaved PARP. To inhibit FSTL1 expression, we purchased three specific siRNAs from Ambion, Life Technologies (Carlsbad, CA, USA). After transfection of siRNAs in NCI-H460 cells as described in Materials and Methods, we tested FSTL1 expression by RT-PCR and real-time RT-PCR. Three kinds of siRNAs that located in different exons inhibited FSTL1 expression (**A, B**). All siRNAs induced cleaved PARP and decreased phosphorylated Erk1/2 as shown by western blotting (**C**) One of siRNAs, si-FSTL1-III that targeted exon 6 and 7 of FSTL1 was commonly used for the experiments.

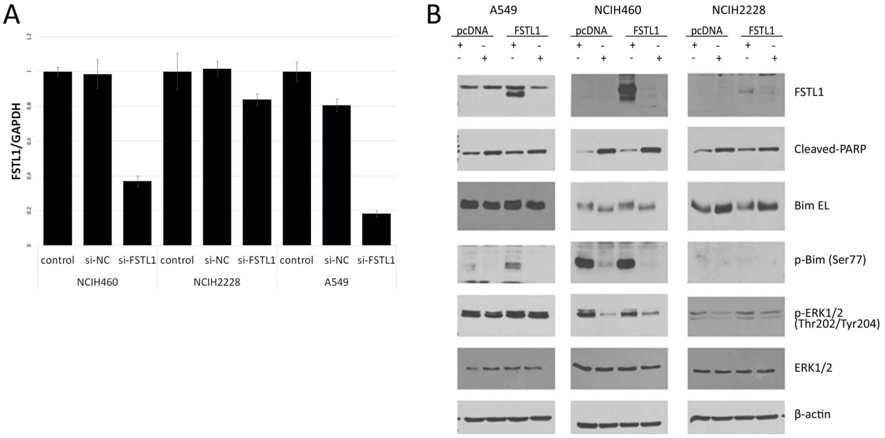

Supplementary Figure S3: The effect of FSTL1 knockdown examined in three different cell lines of lung cancer. Inhibited FSTL1 by siRNA was confirmed by real-time RT-PCR in A549, NCI-H460, and H2228 cell lines. (**A**). We tested the effect of FSTL1 knockdown in cells after transfection of expression vectors. FSTL1 knockdown induced PARP cleavage in three cell lines. The prominent down-regulation of phosphorylated Bim and Erk1/2 were demonstrated in NCI-H460 cells, and decreased phosphorylation of Erk1/2 was detected in all cell lines (**B**).

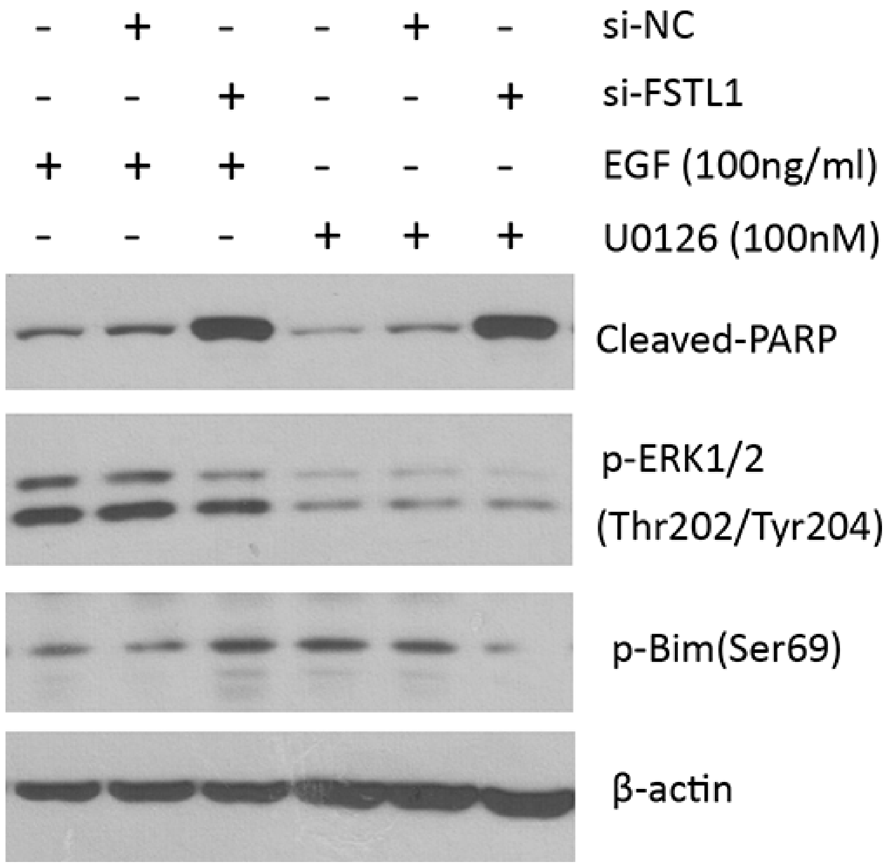

Supplementary Figure S4: The effect of Erk1/2 regulation tested with Mek1/2 inhibitor, U0126 and human EGF. Erk1/2 was inhibited with specific inhibitor of MEK1 and MEK2, U0126 (1, 4-diamino-2, 3-dicyano-1, 4-bis[2-aminophenylthio] butadiene) that was purchased from Selleck Chemicals (Houston, TX, USA). Human Epidermal growth factor (EGF, Sigma-Aldrich, St. Louis, MO, USA) was used to activate Erk1/2. NCI-H460 cells were treated with human EGF or U0126 after transfection of siRNAs. Inactivated Erk1/2 was confirmed by western blotting for phosphorylated Erk1/2 in U0126 treated cells. Cell death was induced by FSTL1-knockdown in human EGF or U0126 treated cells. Decreased phosphorylation of Bim by si-FSTL1 was dominant in U0126 treated cells.

1. Beer DG, Kardia SL, Huang CC, Giordano TJ, Levin AM, Misek DE, Lin L, Chen G, Gharib TG, Thomas DG, Lizyness ML, Kuick R, Hayasaka S, et al. Gene-expression profiles predict survival of patients with lung adenocarcinoma. Nature medicine. 2002; 8:816–824.
2. Bhattacharjee A, Richards WG, Staunton J, Li C, Monti S, Vasa P, Ladd C, Beheshti J, Bueno R, Gillette M, Loda M, Weber G, Mark EJ, et al. Classification of human lung carcinomas by mRNA expression profiling reveals distinct adenocarcinoma subclasses. Proceedings of the National Academy of Sciences of the United States of America. 2001; 98:13790–13795.
3. Garber ME, Troyanskaya OG, Schluens K, Petersen S, Thaesler Z, Pacyna-Gengelbach M, van de Rijn M, Rosen GD, Perou CM, Whyte RI, Altman RB, Brown PO, Botstein D. Diversity of gene expression in adenocarcinoma of the lung. Proceedings of the National Academy of Sciences of the United States of America. 2001; 98:13784–13789.
4. Hou J, Aerts J, den Hamer B, van Ijcken W, den Bakker M, Riegman P, van der Leest C, van der Spek P, Foekens JA, Hoogsteden HC, Grosveld F, Philipsen S. Gene expression-based classification of non-small cell lung carcinomas and survival prediction. PloS one. 2010; 5:e10312.
5. Landi MT, Dracheva T, Rotunno M, Figueroa JD, Liu H, Dasgupta A, Mann FE, Fukuoka J, Hames M, Bergen AW, Murphy SE, Yang P, Pesatori AC, et al. Gene expression signature of cigarette smoking and its role in lung adenocarcinoma development and survival. PloS one. 2008; 3:e1651.
6. Okayama H, Kohno T, Ishii Y, Shimada Y, Shiraishi K, Iwakawa R, Furuta K, Tsuta K, Shibata T, Yamamoto S, Watanabe S, Sakamoto H, Kumamoto K, Takenoshita S, Gotoh N, Mizuno H, et al. Identification of genes upregulated in ALK-positive and EGFR/KRAS/ALK-negative lung adenocarcinomas. Cancer research. 2012; 72:100–111.
7. Selamat SA, Chung BS, Girard L, Zhang W, Zhang Y, Campan M, Siegmund KD, Koss MN, Hagen JA, Lam WL, Lam S, Gazdar AF and Laird-Offringa IA. Genome-scale analysis of DNA methylation in lung adenocarcinoma and integration with mRNA expression. Genome research. 2012; 22:1197–1211.
8. Stearman RS, Dwyer-Nield L, Zerbe L, Blaine SA, Chan Z, Bunn PA, Jr., Johnson GL, Hirsch FR, Merrick DT, Franklin WA, Baron AE, Keith RL, Nemenoff RA, Malkinson AM and Geraci MW. Analysis of orthologous gene expression between human pulmonary adenocarcinoma and a carcinogen-induced murine model. The American journal of pathology. 2005; 167:1763–1775.
9. Su LJ, Chang CW, Wu YC, Chen KC, Lin CJ, Liang SC, Lin CH, Whang-Peng J, Hsu SL, Chen CH and Huang CY. Selection of DDX5 as a novel internal control for Q-RT-PCR from microarray data using a block bootstrap re-sampling scheme. BMC genomics. 2007; 8:140.
10. Talbot SG, Estilo C, Maghami E, Sarkaria IS, Pham DK, P Oc, Socci ND, Ngai I, Carlson D, Ghossein R, Viale A, Park BJ, Rusch VW and Singh B. Gene expression profiling allows distinction between primary and metastatic squamous cell carcinomas in the lung. Cancer research. 2005; 65:3063–3071.
11. Wachi S, Yoneda K and Wu R. Interactome-transcriptome analysis reveals the high centrality of genes differentially expressed in lung cancer tissues. Bioinformatics. 2005; 21:4205-4208.
12. Wei TY, Juan CC, Hisa JY, Su LJ, Lee YC, Chou HY, Chen JM, Wu YC, Chiu SC, Hsu CP, Liu KL and Yu CT. Protein arginine methyltransferase 5 is a potential oncoprotein that upregulates G1 cyclins/cyclin-dependent kinases and the phosphoinositide 3-kinase/AKT signaling cascade. Cancer science. 2012; 103:1640–1650.
13. Weiss J, Sos ML, Seidel D, Peifer M, Zander T, Heuckmann JM, Ullrich RT, Menon R, Maier S, Soltermann A, Moch H, Wagener P, Fischer F, Heynck S, Koker M, Schottle J, et al. Frequent and focal FGFR1 amplification associates with therapeutically tractable FGFR1 dependency in squamous cell lung cancer. Science translational medicine. 2010; 2:62ra93.
